# Antimicrobial potency, prevention ability, and killing efficacy of daptomycin-loaded versus vancomycin-loaded β-tricalcium phosphate/calcium sulfate for methicillin-resistant *Staphylococcus aureus* biofilms

**DOI:** 10.3389/fmicb.2022.1029261

**Published:** 2022-11-03

**Authors:** Xin Zhang, Peng Chen, Hao-yang Wan, Run-jiu Zhu, Yue Zhou, Ming-rui Song, Nan Jiang, Bin Yu

**Affiliations:** ^1^Division of Orthopaedics and Traumatology, Department of Orthopaedics, Nanfang Hospital, Southern Medical University, Guangzhou, China; ^2^Department of Orthopaedics and Traumatology, Wuyi Hospital of Traditional Chinese Medicine, Jiangmen, China; ^3^Department of Orthopaedics, Hainan General Hospital, Hainan Hospital Affiliated to Hainan Medical University, Haikou, China; ^4^Guangdong Provincial Key Laboratory of Bone and Cartilage Regenerative Medicine, Nanfang Hospital, Southern Medical University, Guangzhou, China; ^5^School of Nursing, Jiangmen Chinese Medicine College of Guangdong Province, Jiangmen, China

**Keywords:** daptomycin, vancomycin, MRSA, biofilm, implant-associated infection, *in vitro* study

## Abstract

Growing evidence has shown that the efficacy of systemic administration of daptomycin for the treatment of methicillin-resistant *Staphylococcus aureus* (MRSA)-related infections is satisfactory. However, the clinical efficacy of the local administration of daptomycin for the management of osteoarticular infections remains unclear. This *in vitro* study compared the efficacy of daptomycin and vancomycin against MRSA biofilms. The elution kinetics of daptomycin and vancomycin, combined with gentamicin and loaded with either β-tricalcium phosphate/calcium sulfate or calcium sulfate, in the presence of MRSA infection, was assessed. Their efficacy in preventing biofilm formation and killing pre-formed biofilms was assessed using colony-forming unit count and confocal laser scanning microscopy. In addition, the efficacy of daptomycin, vancomycin, and gentamicin in prophylaxis and eradication of MRSA biofilms was also evaluated. Daptomycin + gentamicin and vancomycin + gentamicin displayed similar antimicrobial potency against MRSA, by either β-tricalcium phosphate/calcium sulfate or calcium sulfate. In the prevention assays, both daptomycin + gentamicin and vancomycin + gentamicin showed similar efficacy in preventing bacterial colony formation, with approximately 6 logs lower colony-forming units than those in the control group at both 1 and 3  days. The killing effect on pre-formed biofilms showed significant decreases of approximately 4 logs at 1 and 3  days following treatment with daptomycin + gentamicin and vancomycin + gentamicin. In addition, the confocal laser scanning microscopy results support the colony-forming unit data. Moreover, single use of vancomycin and gentamicin showed similar efficacies in preventing and killing MRSA biofilms, both of which were better than that of gentamicin. Our study demonstrated that vancomycin + gentamicin and daptomycin + gentamicin loaded with β-tricalcium phosphate/calcium sulfate or calcium sulfate showed similar prophylactic and killing effects on MRSA biofilms, implying a potential indication of local administration daptomycin for the treatment of MRSA-associated osteoarticular infections, especially if vancomycin administration presents limitations.

## Introduction

With the arrival of our aging society and an increasing number of patients with extremity fractures, the clinical use of artificial joints and internal fixations is increasing. The most challenging complications following joint replacement and fracture fixation procedures are implant-associated infections (IAIs), including periprosthetic joint infections (PJIs) and fracture-related infections (FRIs) (Trampuz and Widmer, 2006; Horton et al., 2021). According to recent studies, the cumulative incidence after primary hip or knee arthroplasty is 0.5% in the first year and 1.4% within 10 years, whereas the incidence of FRI ranges from 0.4 to 16.1%, with an average of 5%, and up to 30% following open fractures (Beam and Osmon, 2018; Foster et al., 2020; Jiang et al., 2021). Despite advances in surgical techniques, IAIs still pose great challenges to orthopedic surgeons, owing to their high risk of disability, mortality, and infection recurrence.

*Staphylococcus aureus* (*S. aureus*) is the main causative agent of IAIs, and methicillin-resistant *S. aureus* (MRSA) accounts for approximately 25–50% of the isolates (Gatti et al., 2022). They can form difficult-to-eradicate biofilms, which is the primary reason for infection recurrence (Arciola et al., 2018; Mandell et al., 2019). The frequently used strategy to remove bacterial biofilms is radical wound debridement with pulsed lavage (PL), followed by local implantation of antibiotics (Zimmerli, 2014; Depypere et al., 2020). PL is used to mechanically disrupt and eliminate bacterial biofilms in bones, soft tissues, and implant components. However, an *in vitro* study showed that PL could remove approximately 90% of the biofilms from artificial joint materials (Knecht et al., 2018). Therefore, in addition to physical removal, the local use of microbeads containing antibiotics is another effective way to control bacterial reproduction that may remain or subsequently enter the surgical site (Foster et al., 2020; Aggarwal et al., 2022). Materials that are commonly used to imbed the antibiotics are classified as non-absorbable [such as polymethyl methacrylate (PMMA)], or absorbable materials, [such as high-purity calcium sulfate (CS)]. Previous studies have indicated that they can be administered for both the treatment and prophylaxis of IAIs (Howlin et al., 2015; van Vugt et al., 2019; Aggarwal et al., 2022).

Initially, gentamicin (GEN, G) was locally administered to treat IAIs; however, with the increasing rate of antibiotic resistance, it was less frequently administered alone. Tobramycin (TOB, T) and vancomycin (VAN, V) are often administered for MRSA-related infections. However, increasing reports have indicated resistance to TOB (Zalavras et al., 2004; Jackson et al., 2021; Sumrall et al., 2021). Likewise, although VAN has been considered the “backbone” against MRSA-related infections, serious concerns have been raised regarding its limitations, such as the low therapeutic index, limited tissue penetration, slow bactericidal activity, as well as the increasing reports of resistance and treatment failure (Bruniera et al., 2015). For these reasons, the search for therapeutically effective alternative drugs, especially those that can replace their local use in IAIs, is critical (Stryjewski and Corey, 2009).

Many alternative drugs had been approved by the U.S. Food and Drug Administration (FDA) for the treatment of MRSA-related infections (Micek, 2007), such as linezolid, daptomycin (DAP, D), and tigecycline. DAP, a novel cyclic lipopeptide drug, was approved by the FDA in 2003 for the treatment of complicated skin and skin structure infections (cSSSIs), right-sided infective endocarditis, bacteremia following cSSSIs, and right-sided infective endocarditis. DAP has also been evaluated for IAIs treatment (Heidary et al., 2018). Several experimental studies comparing the efficacy of DAP and VAN have shown that DAP may have more advantages than VAN such as greater bone penetration and longer half-life. Most initial studies and clinical data proved that DAP is more efficacious than VAN in certain situations (e.g., with lower mortality and nephrotoxicity) for intravenous use in the treatment of bacteremia due to MRSA infections (Moenster et al., 2012; Moise et al., 2016). Further *in vivo* and *in vitro* experiments have demonstrated good outcomes of DAP in the treatment of osteomyelitis (Lamp et al., 2007). However, studies focusing on the local administration of DAP for the treatment of IAIs remain limited.

Concerning the local administration of antibiotics for the treatment of orthopedic infections, VAN in combination with GEN, loaded by carriers to achieve a persistent release effect, is more frequently selected clinically (Zalavras et al., 2004; Jackson et al., 2021). Thus, this study aimed to assess the efficiency of D + G and V + G loaded with two biomaterials, namely, β-tricalcium phosphate/calcium sulfate (β-TCP/CS) and CS, against bacterioplankton or biofilms of MRSA. One advantage of such antibiotic vehicles is that they are completely biodegradable, and thus do not require a second surgical removal. Additionally, β-TCP/CS has an osteoconductive effect and provides bone scaffolding (Urban et al., 2007; Fernandez de Grado et al., 2018; Zhao et al., 2020). Here, we analyzed the release characteristics of DAP and compared the efficiency of DAP with that of VAN in preventing and killing MRSA biofilms, concerning a previously established *in vitro* biofilm model (Jiang et al., 2021). Through these experiments, we provide a theoretical basis for the local administration of DAP for the treatment of IAIs in clinical settings.

## Materials and methods

### Bacterial strains and growth media

ATCC43300, an MRSA strain that has been shown to form biofilms on polymer surfaces, was selected for this study (Tan et al., 2012). The MRSA strain, provided by our laboratory, was cultured in trypsin-soybean broth (TSB; Pythonbio, Guangdong, China) overnight at 37°C with shaking at 200 rpm in shaking conditions. The minimum inhibitory concentrations (MICs) of D, V, and G were determined using the microfluidic dilution method (Wiegand et al., 2008).

### Preparation of antibiotic-loaded beads (ALBs)

ALBs were prepared using Genex and Stimulan Rapid Cure products (Biocomposites Ltd., Staffordshire, United Kingdom). Genex is a synthetic biphasic material consisting of β-TCP and CS in a 1:1 weight ratio, whereas stimulant is a high-purity CS product. The mixing ratios for the D + G and V + G groups were 1,000 mg DAP (Hengrui Ltd., Jiangsu, China) with 1,000 mg GEN (Tianxin Ltd., Guangdong, China) 240 mg, and VAN (Eli Lily & Co., Indiana, United States) with gentamicin 240 mg, respectively, mixed with 10 ccs of Genex or Stimulan powder (Weiss et al., 2009; Kaplan et al., 2012). Similarly, the mix ratios for groups D, V, and G alone were DAP 1,000 mg, VAN 1,000 mg, and GEN 240 mg, with Genex or Stimulan powder per 10 ccs. A combination of sterile normal saline (NS), the respective antibiotic powder, and β-TCP/CS or CS powder was hand-mixed under aseptic conditions with dry powder and water for 30 s until a smooth paste was produced. The smooth paste was pressed in a flexible mold containing 4.8 mm wide hemispherical depressions placed at 20°C. After hardening for 24 h, the beads were released from the mold before use (Knecht et al., 2018).

### Antibiotic-elution potency and duration for inhibition of planktonic bacteria

The modified Kirby–Bauer test (KBT) was used to determine the potency of ALBs over time (Boyle et al., 1973). Overnight cultures of MRSA (ATCC43300) were diluted to 1% in TSB medium, 100 μl of the diluted medium was applied to tryptic soy agar (TSA, Pythonbio, Guangdong, China) agar plates, and ALBs with D + G or V + G were placed centrally on top of the agar using aseptic forceps. Petri dishes were incubated at 37°C and 5% CO_2_ for 24 h, followed by the analysis and recording of zones of inhibition (ZOIs). The beads were transferred to a new bacterial agar dish. The maximum diameter was measured every 24 h using a straightedge and recorded, and the process was repeated daily until the ZOIs measured diameter of less than 0.6 cm. Then, the ZOIs were calculated using the equation *A* = *πr*^2^, where *A* is the area of the circle, *r* is the radius (half of the diameter), and *π* is a constant (approximately equal to 3.142). This area is used instead of the diameter because the inhomogeneity of the beads sometimes leads to irregularly shaped ZOIs.

### Prevention of biofilm formation and killing efficacy of established biofilms

ALBs and unloaded antibiotic beads (two beads per well) were placed in 12-well plates (Corning, NY, United States) and confocal dishes with a diameter of 35 mm (Corning, NY, United States). For biofilm prevention experiments, the bacteria were diluted to a concentration of approximately 10^6^ colony-forming unit (CFU)/ml and each well was inoculated with 2 ml of overnight culture and incubated at 37°C and 50 rpm on a shaker. The bacteria were incubated for 1 or 3 days, with the culture medium containing 2 ml of 10^6^ CFU/ml of fresh bacteria, changed every 24 h. For the killing assay, 2 ml of 10^6^ CFU/ml MRSA was inoculated per well and incubated at 37°C and 50 rpm on a shaker for 3 days to allow the establishment of biofilms, with 1 ml of the fresh medium changed daily. Then, two ALBs or unloaded antibiotic beads were added to each well with fresh medium, and the medium was changed every 24 h for 3 days.

### Viable CFUs

Each well was rinsed twice with 2 ml of phosphate-buffered saline (PBS; Gibco, MD, United States) to remove planktonic cells. The surface of each well was scraped using a cell scraper (Corning, NY, United States), which was then placed in 1 ml PBS, rotated, and shaken for 20 s to homogenize the biofilm bacteria, serially diluted ten times, and plated on TSA agar plates using the drop plate method for a total of three replicates (Herigstad et al., 2001). The control groups were the groups without beads (control 1, C1) and the groups with blank beads (without antibiotics; control 2, C2).

### Confocal laser scanning microscopy (CLSM) and fluorescence microscope

CLSM and fluorescence microscopy were performed to examine biofilms grown on 35-mm confocal dish plates (Zeiss LSM 980 and ZEISS Axio Imager 2, Germany). The SYTO9/Propidium iodide (PI) live and dead bacterial kit (Pythonbio, Guangdong, China) was used to visualize the biofilms. Each dish was rinsed with PBS and stained with 1.5 μl SYTO9 + 1.5 μl PI per 1 ml PBS or NS for 20 min, according to the manufacturer’s instructions. The plate was then gently rinsed again with PBS or sterile NS, and 1 ml of PBS was added to the wells before observation by CLSM or fluorescence microscopy.

### Statistical analysis

Statistical analyses were performed using GraphPad Prism 9.0 (GraphPad Software, United States). The CFU data were firstly log_10_ transformed, and data were expressed as mean ± standard deviation (SD). Student’s *t*-test or one-way analysis of variance (ANOVA) was used to compare differences in CFUs between two or more groups. For one-way ANOVA, *post hoc* multiple comparisons were conducted using Sidak’s test. Statistical significance was set at *p* ≤ 0.05.

## Results

### Antibiotic MICs, elution potency, and duration for inhibition of planktonic bacteria

**Figure 1:**
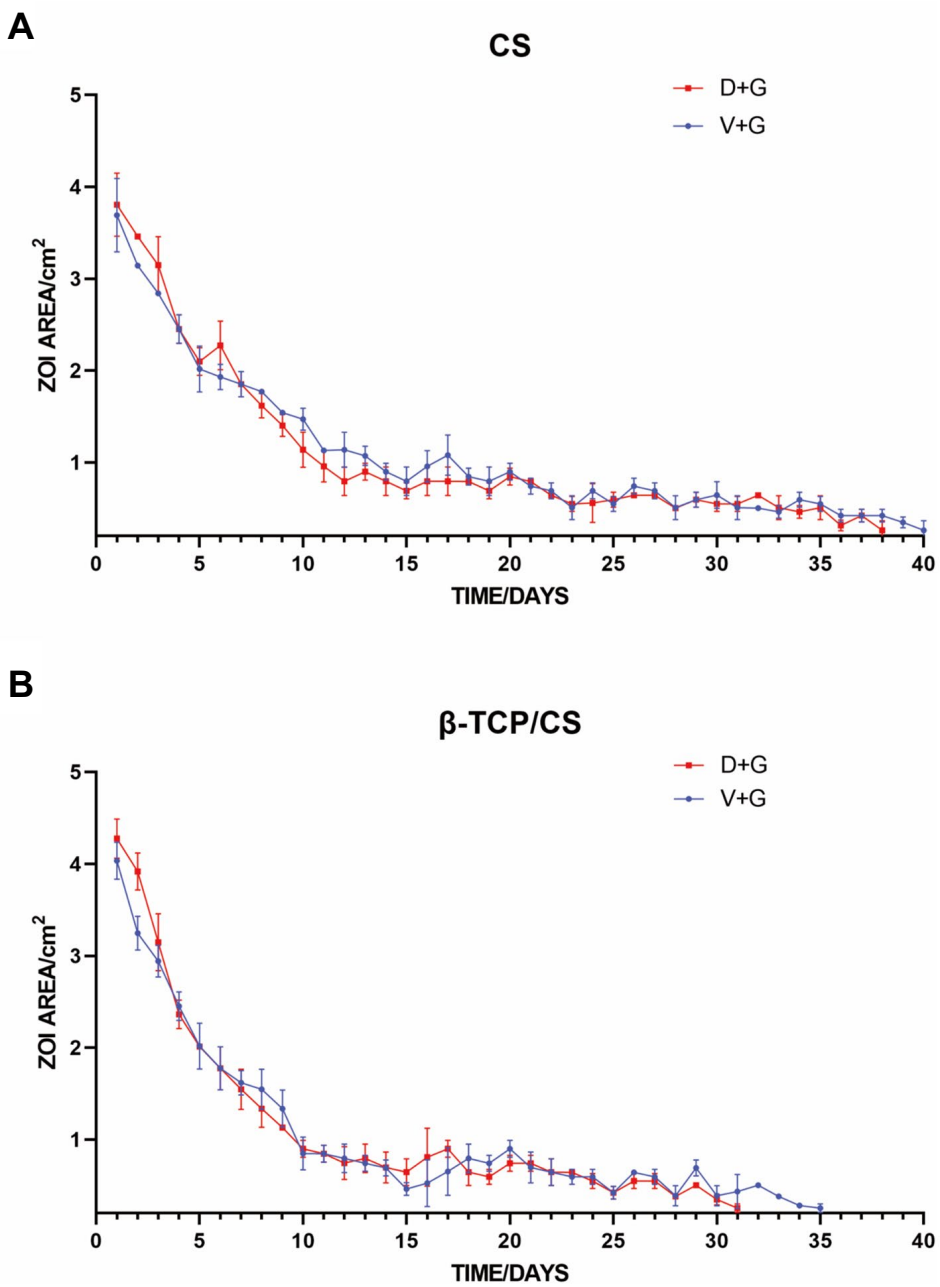
Repeated zones of inhibition (ZOIs) of MRSA, showing the size of the ZOIs (cm^2^) produced by antibiotic-loaded CS **(A)** and β-TCP/CS **(B)** beads over time. Experiments were performed using three replicates, and data are expressed as mean ± standard deviation (SD).

The minimum inhibitory concentrations (MICs) of D, V, and G for ATCC43300 were 0.5, 1, and 32 mg/l, respectively. MRSA was sensitive to both D + G and V + G piggybacking on different materials, with D + G and V + G exhibiting potencies of 31 and 35 days from β-TCP/CS beads and 38 and 40 days from CS beads, respectively. V + G and D + G showed similar potency effects between the two materials, both showing an initial 10-day burst of potency release followed by a flat release until the end of the measurement (Figure 1).

### Prevention of biofilm formation and killing efficacy of established biofilms

**Figure 2:**
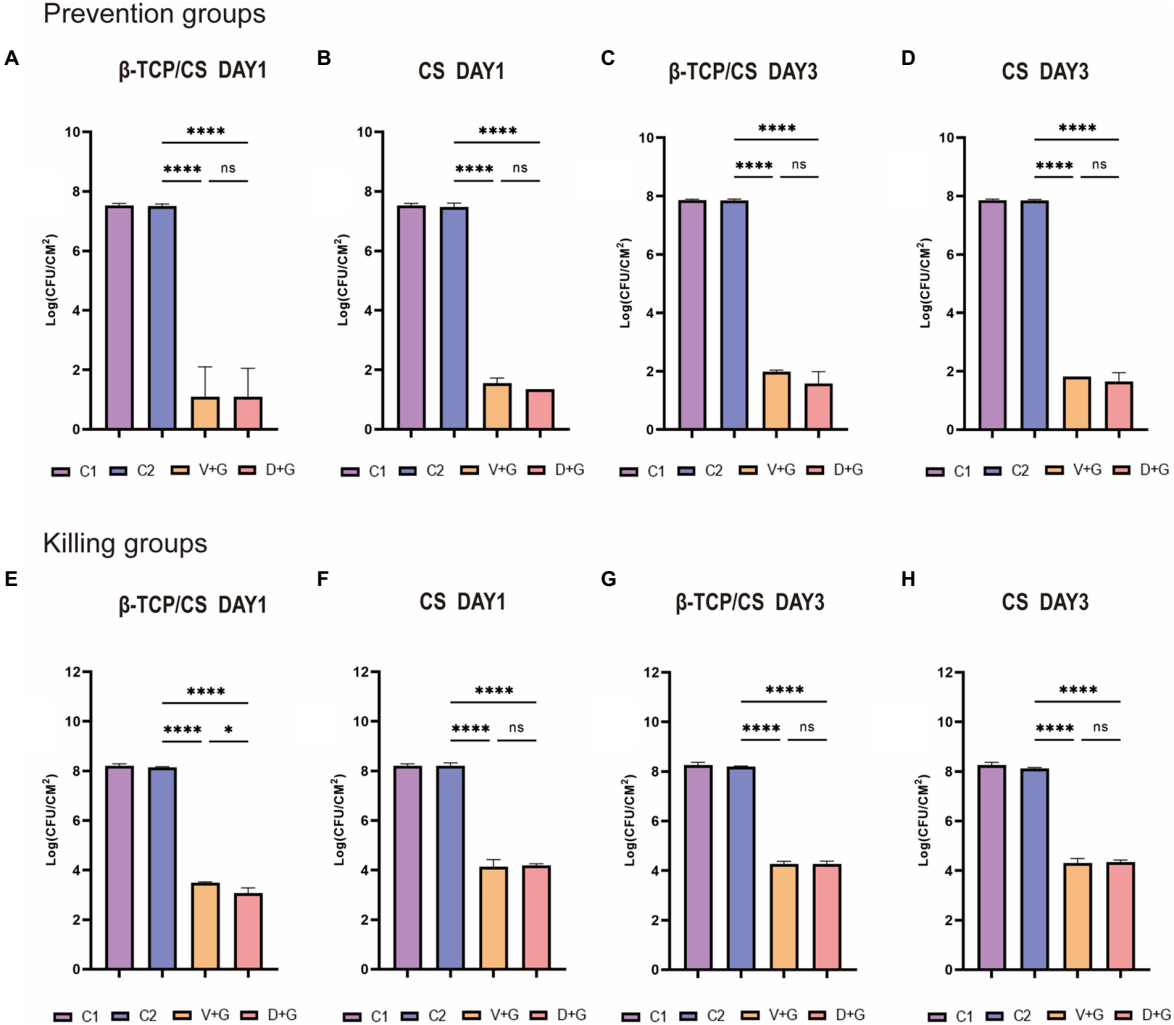
Methicillin-resistant *Staphylococcus aureus* (MRSA) CFUs were counted on days 1 and 3 between the prevention and killing groups. Data are expressed as mean ± standard deviation (SD). Prevention groups **(A–D)**; killing groups **(E–H)**; C1, control groups without beads; C2, control groups with blank beads (without antibiotics). ns, not significant; ^*^p < 0.05, ^****^p < 0.001.

Bacterial quantification of the colonies in each experimental group was performed using the CFU counting method. In the prophylactic assays (as shown in Figures 2A–D), the number of MRSA biofilms in the control groups [including groups without beads (C1) and groups with blank beads (C2)] was approximately 10^7^ to 10^8^ CFU/cm^2^ at 1 and 3 days. Significant differences were found in the CFUs among the four groups at 1 day, suggesting that CFUs in the D + G and V + G groups were approximately 6 logs less live MRSA colonies than those in the control groups (*p* < 0.001), either by β-TCP/CS or by CS beads. No significant difference was observed in CFUs between D + G and V + G by post-hoc multiple comparisons, either by β-TCP/CS or CS beads (Figures 2A,B). Similar outcomes were observed 3 days after the respective interventions (Figures 2C,D).

Regarding the outcomes of the killing assays, after consecutive culturing for 3 days, the number of MRSA colonies in the control groups had grown over 10^8^ CFU/cm^2^ (Figures 2E–H). Likewise, significant differences were also identified among the four groups at 1 day, demonstrating that CFUs in the D + G and V + G groups were approximately 4–5 logs fewer live MRSA colonies than those in the control groups (*p* < 0.001), either by β-TCP/CS or CS beads (Figures 2E,F). Similar outcomes were also observed after 3 days (Figures 2G,H). Concerning *post-hoc* multiple comparisons, results showed that aside from the slightly higher CFUs in V + G compared to D + G at 1 day by β-TCP/CS beads (*p* < 0.05), no significant differences were noted among other comparisons, either at 3 days by β-TCP/CS beads, or at 1 or 3 days by CS beads.

**Figure 3:**
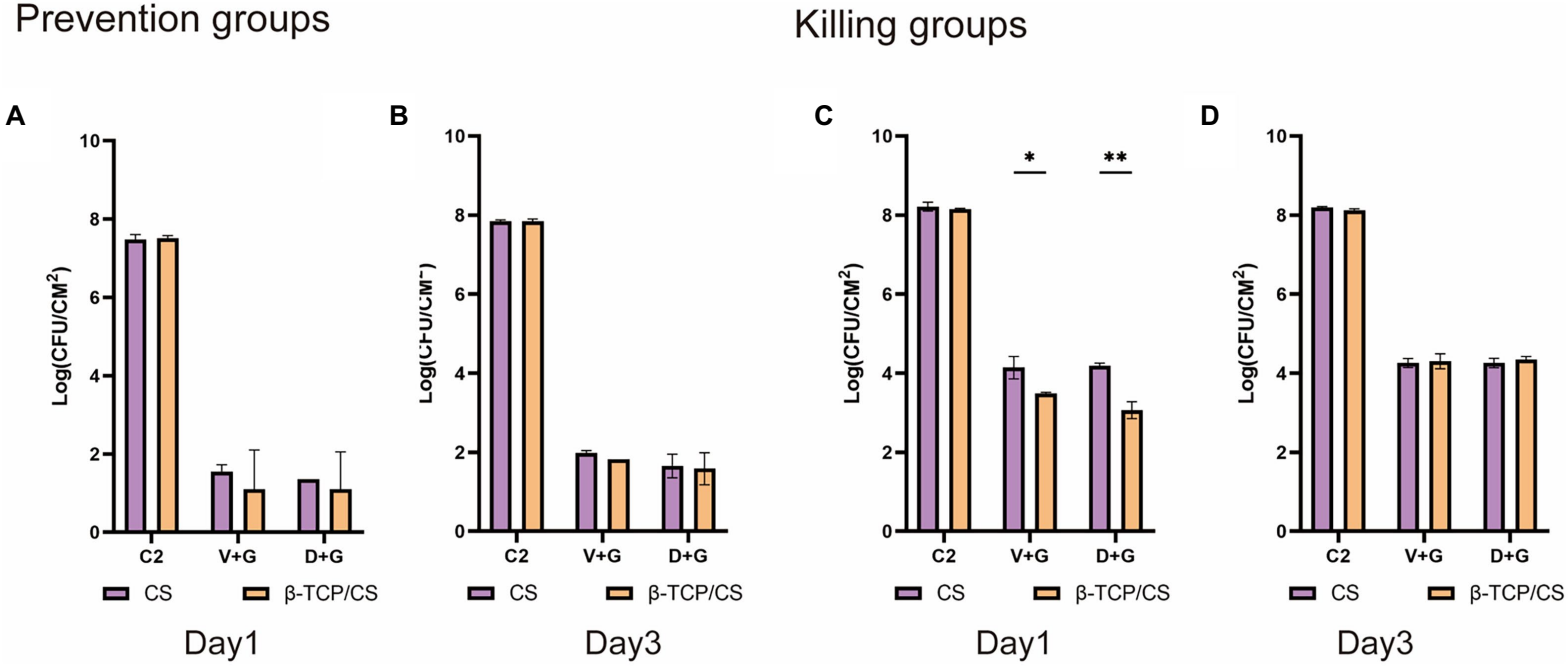
Comparisons of CFUs of MRSA after treatment with β-TCP/CS or CS beads in the prevention **(A,B)** and killing groups **(C,D)**. Data are expressed as mean ± standard deviation (SD). C2, control groups with blank beads (without antibiotics). ^*^p < 0.05, ^**^p < 0.01.

Concerning potential differences between the two antibiotic carriers, no significant differences were found in CFUs between β-TCP/CS and CS in the prevention experiments, either at 1 or 3 days (Figures 3A,B). For the killing experiments, β-TCP/CS revealed significantly decreased CFUs compared to CS at 1 day, either loaded with D + G or V + G. At day 3, no differences were found between the two materials (Figures 3C,D). Regarding the outcomes of the single antibiotic groups, as shown in Supplementary Figures 1A–D, in the prophylaxis assays, significant differences were found in the CFUs among the four different intervention groups, at 1 or 3 days, either by β-TCP/CS or by CS beads. Post-hoc comparisons showed that the mean CFUs of the G group were significantly higher than those of the D and V groups, at 1 or 3 days, either by β-TCP/CS or by CS (*p* < 0.001). Similar tendencies were observed in the killing groups (Supplementary Figures 1E–H).

### CLSM and fluorescence microscope

**Figure 4:**
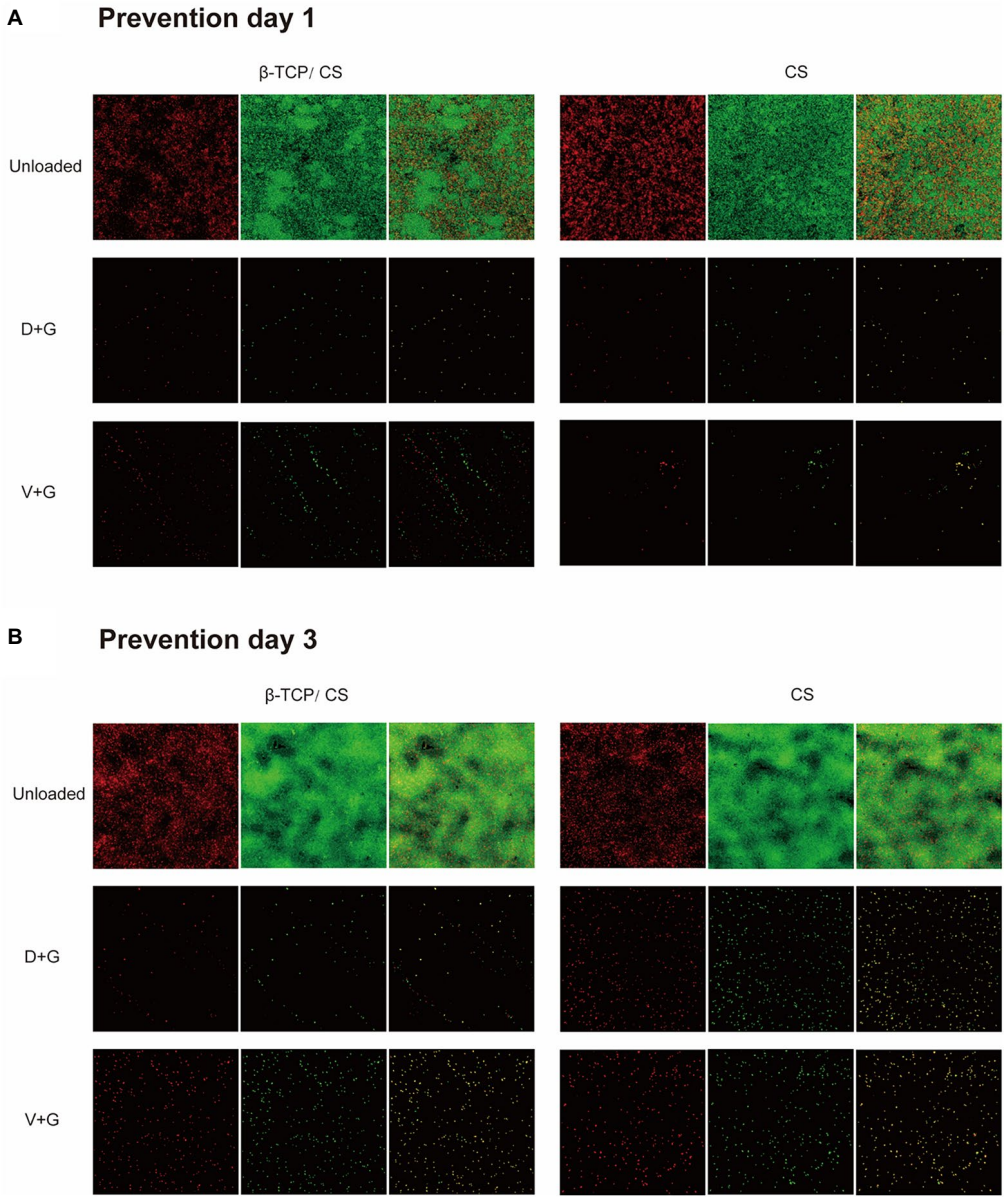
Prevention of MRSA biofilm formations on glass surfaces of confocal dish culture plates at days 1 **(A)** and 3 **(B)**, as observed *via* CLSM. The biofilms were stained with live and dead stain, where green represents live and red represents dead bacterial cells within the biofilms. The third column in each group is a merge of the previous two columns (live and dead stains).

CLSM and fluorescence microscopy were performed to assess the efficacy of different antibiotics, either alone or in combination, for the prevention and eradication of MRSA biofilms. In the prevention assays (as shown in Figure 4A), a visible MRSA biofilm was established in the control group on day 1, with a roughly uniform multilayer structure and a dense accumulation of live and dead bacteria. The majority of the MRSA were live and displayed a green signal. After exposure to antibiotics, an obvious decrease in the number of MRSA colonies was noted in both the D + G and V + G groups without multilayer accumulation. After 3 days of exposure, an increased density of MRSA biofilms was observed in the control group. In contrast, in the antibiotic groups, the number of MRSA colonies remained much lower than that of the control group, although there was a slightly increased density of signals compared to those on day 1 (Figure 4B).

**Figure 5:**
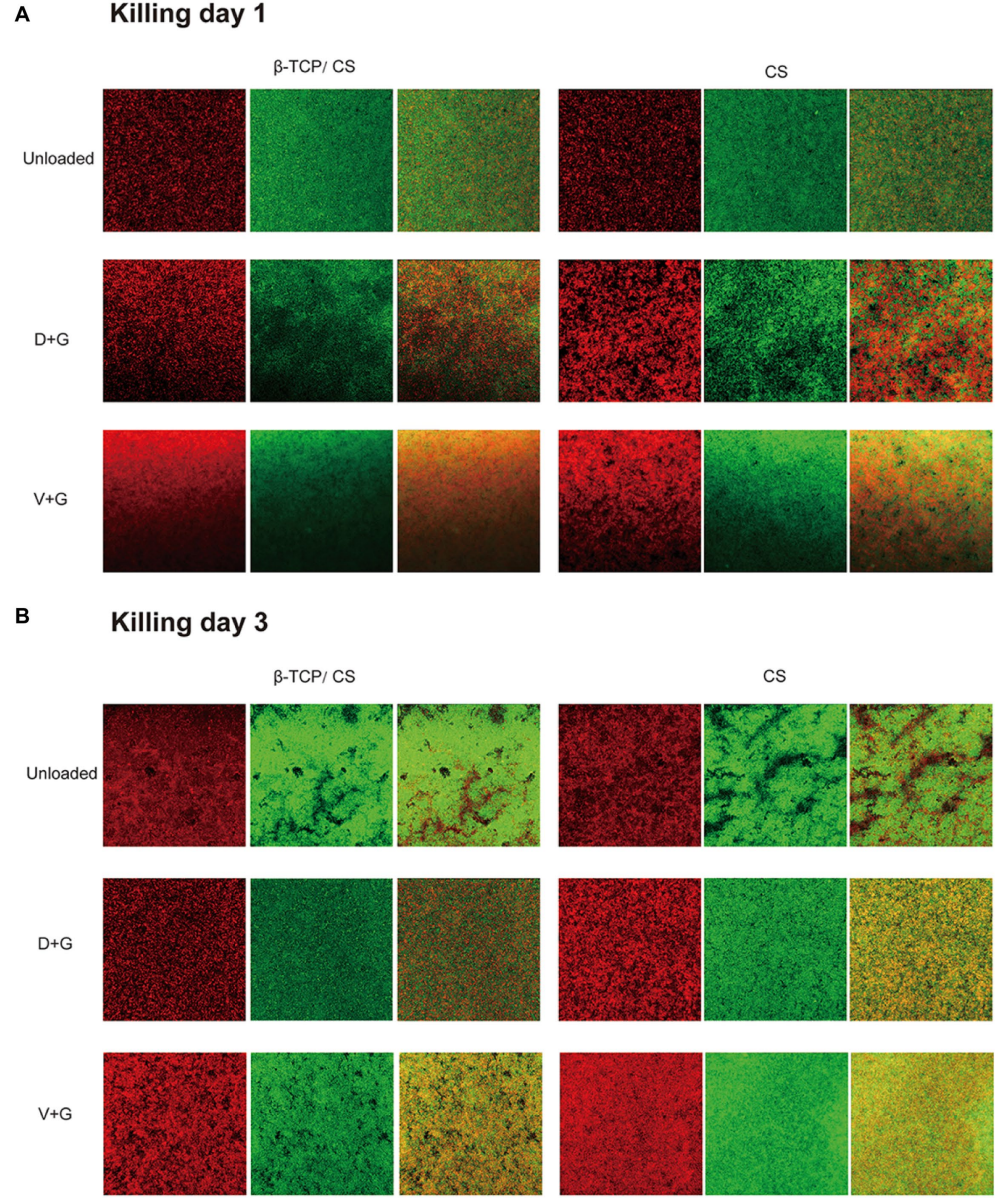
Killing of biofilm formed by MRSA on glass surfaces of confocal disc culture plates on days 1 **(A)** and 3 **(B)** after biofilm formation, as observed *via* CLSM. Biofilms were stained with live and dead stains; green, live bacterial cells within biofilms; red, dead bacterial cells within biofilms. The third column in each group is a merge of the previous two columns (live and dead stains).

In the killing assays, all groups were exposed to different interventions after 72 h of mature biofilm formation. As can be seen in Figures 5A,B, more dense accumulations of live and dead bacteria were found in the control group on both days 1 and 3, with more dead bacteria interspersed in the multilayer MRSA colonies than in the respective prevention groups. Nonetheless, most of the bacteria remained in the control group. In contrast, dead bacteria were predominant in the antibiotic-treated groups. In addition, compared with those in the V + G groups, the number of viable bacteria in the D + G groups appeared to be lower on days 1 and 3.

As for the single antibiotic groups, the Supplementary Figures 2, 3 showed that compared with those in the control groups, reductions in fluorescence signals were observed in the D, V, and G groups in both prophylactic and killing assays. Similar outcomes were observed in both the β-TCP/CS and CS groups.

## Discussion

Despite great advances in medical techniques, IAIs remain the most challenging complication of orthopedic surgery. Bacterial biofilms play a critical role in the occurrence, development, and deterioration of infections and influence treatment efficacy (Stoodley et al., 2011). Thus, effective prevention and eradication of biofilms are of great clinical significance. Radical debridement, followed by local implantation of antibiotics, is the mainstay of IAIs therapy. Therefore, selection of sensitive antibiotics, either alone or in combination, is crucial (Knecht et al., 2018; Aggarwal et al., 2022). Currently, *S. aureus* is one of the most frequently detected microorganisms causing IAIs, with MRSA accounting for a large percentage. In the past few decades, VAN has always been the first-line antibiotic against MRSA; however, with the increasing incidence of adverse events and increasing reports of resistance and treatment failure, the identification of alternative antibiotics is essential (Micek, 2007). Here, we focused on DAP and compared its efficacy with that of VAN against MRSA biofilms. As VAN is commonly administered in combination with GEN for the clinical management of IAIs, aside from single antibiotics, we also evaluated the efficiency of combinations of antibiotics, including D + G and V + G, for the prevention and eradication of MRSA biofilms.

We found that D + G and V + G shared similar release potency and efficacy against MRSA biofilms, both of which exhibited an effective antibiotic release for 30–40 days, with a rapid release in the first 10 days, followed by a slow and persistent release until the end. Previous studies have also indicated that DAP possesses a release rate similar to that of VAN in PMMA (Hall et al., 2004; Kollef, 2007). In addition, we also noted that antibiotics loaded with β-TCP/CS had a shorter duration of release than those loaded with CS, which may be associated with the fact that β-TCP has a higher porosity, facilitating the release of antibiotics (Zhao et al., 2020). In the prevention assays, the results revealed that β-TCP/CS or CS microspheres carrying D + G and V + G could effectively prevent biofilm formation on days 1 and 3, with significantly fewer CFUs (a reduction of approximately 6 logs). As for the killing experiments, such a reduction reached 4 logs on both days 1 and 3 after implantation of the antibiotics, with similar efficacy between D + G and V + G, demonstrating the possibility of DAP as an alternative antibiotic for local administration against MRSA biofilms.

Although combination antibiotic regimens are frequently administered in clinical practice to widen the antibacterial spectrum, a single administration of such antibiotics should be conducted to better evaluate their efficacy against biofilms. Thus, we assessed the efficacy of single antibiotics D, V, and G in the prevention and eradication of MRSA biofilms. Based on the MIC outcomes, this MRSA strain appeared to be resistant to G, which still displayed good efficiency in the present study. One underlying factor may be related to the localized presence of high antibiotic concentrations rather than an oral or intravenous scenario, where drug concentrations may be lower and fluctuate due to pharmacokinetics. Our results demonstrated that both D and V may possess better capacities than G to prevent and kill MRSA biofilms, which was supported by fluorography. It should be noted that such different effects may also be caused by higher doses of D (1,000 mg) and V (1,000 mg) than that of G (240 mg) in our study.

Growing evidence indicates that DAP possesses fine bactericidal activity against *staphylococci* in both *in vitro* and *in vivo* studies. In an *in vitro* study (Fuchs et al. 2002), Fuchs et al. analyzed the MICs and minimum bactericidal concentrations (MBCs) of DAP, VAN, linezolid, and quinupristin–dalfopristin against 108 *Staphylococcus* isolates, and the results revealed that daptomycin was bactericidal against most strains and had the most rapid bactericidal activity. In a recent systematic review and meta-analysis (Samura et al., 2022), Samura et al. compared the efficacy of DAP with VAN in adult patients with MRSA bacteremia with VAN MIC exceeding 1 μg/ml. Based on the synthesis of seven studies of 907 patients, they found that compared with VAN, DAP was related to significantly reduced mortality and a higher treatment success rate, especially for patients with intermediate- and high-risk bacteremia sources. Although previous studies have indicated the satisfactory efficacy of DAP against *Staphylococcus* isolates, especially MRSA, most of these were obtained *via* intravenous administration. Currently, the number of studies focusing on local administration of DAP is limited. Several reports have analyzed the efficacy of DAP loaded with PMMA, and the outcomes showed its good antimicrobial capacity without altering the structural characteristics in terms of its release potency (Rosslenbroich et al., 2012; Tan et al., 2012; Ferreira et al., 2015). While our study found that aside from PMMA, DAP can be loaded by β-TCP/CS and CS, and both showed good outcomes in the prevention and eradication of MRSA biofilms. In addition to its antimicrobial capacity, DAP also has advantages such as lower mortality and relapse rates, better tolerability, and fewer adverse events (Lamp et al., 2007; Moenster et al., 2012; Moise et al., 2016; Schweizer et al., 2021). Although DAP has advantages, its wide application remains limited because of its high cost and the uncertainty regarding the dose for pediatric patients under 12 years (Heidary et al., 2018).

Our study has some limitations. First, our experiment was conducted using only in the *vitro* test setup. *In vivo* factors, such as blood flow, movement, protein binding, and involvement of immune cells, may lead to alterations in antibiotic activity. In addition, the model we used in the present study was initially developed for catheter-related blood infections that often grow biofilms at the solid–liquid interface (bottom of the microwell) or the solid–liquid-air interface (wall of the microwell), whereas biofilms in orthopedic IAIs are formed at the device-tissue interface. Thus, the current model may not fully reflect the status of biofilms during IAIs. Second, in the simulated biofilm prevention and killing experiments, the tests were only conducted on days 1 and 3, which is insufficient, and a longer culture time is warranted to better evaluate the efficacy of different antibiotics. Third, we selected only the standard MRSA strain ATCC43300 for the experiment. Different clinical MRSA strains should be used to comprehensively assess the efficacy of DAP against MRSA. Finally, the concentrations (mix ratios) of antibiotics with β-TCP/CS or CS in our study were set, with respect to clinical use, and the optimal DAP concentration remains uncertain. Therefore, subsequent *in vitro* and *in vivo* experiments should be performed to evaluate the efficacy and safety of different concentrations of local DAP for the management of IAIs. Despite these limitations, our study is valuable, as it provides preliminary evidence regarding the local administration of DAP against MRSA biofilms, providing an alternative selection of antibiotics for the treatment of IAIs.

Our experiments demonstrated that D + G loaded with β-TCP/CS and CS displayed similar efficacy to V + G in preventing the formation of, as well as killing, MRSA biofilms. Although VAN remains the first-line agent for the treatment of IAIs, DAP may be a suitable and effective alternative when VAN resistance develops or patients cannot tolerate VAN.

## Data availability statement

The original contributions presented in the study are included in the article/Supplementary material, further inquiries can be directed to the corresponding authors.

## Author contributions

NJ and BY: conceptualization, writing-review and editing, supervision, project administration, and funding acquisition. XZ, NJ, and BY: methodology. XZ, PC, and BY: validation. XZ, H-yW, and NJ: formal analysis. XZ, PC, H-yW, R-jZ, and M-rS: investigation. YZ and R-jZ: data curation. XZ, PC, and NJ: writing-original draft preparation. All authors contributed to the article and approved the submitted version.

## Funding

This study was funded by the National Natural Science Foundation of China (Grant Nos. 81830079, 82172197 and 82272517).

## Conflict of interest

The authors declare that the research was conducted in the absence of any commercial or financial relationships that could be construed as a potential conflict of interest.

## Publisher’s note

All claims expressed in this article are solely those of the authors and do not necessarily represent those of their affiliated organizations, or those of the publisher, the editors and the reviewers. Any product that may be evaluated in this article, or claim that may be made by its manufacturer, is not guaranteed or endorsed by the publisher.

## Supplementary material

SUPPLEMENTARY FIGURE 1 Methicillin-resistant *Staphylococcus aureus* (MRSA) colony forming units (CFUs) were counted on days 1 and 3 between the prevention and killing groups. The data are expressed as mean ± standard deviation (SD). Prevention groups: **(A–D)**; killing groups: **(E–H)**. C refers to the control group with blank beads (without antibiotics); ns: not significant, ^****^*p* < 0.001.

SUPPLEMENTARY FIGURE 2 Prevention of MRSA biofilm formation on the glass surface of confocal dish culture plate at days 1 **(A)** and 3 **(B)** by fluorescence microscopy. The biofilms were stained with live and dead stains, where green represents live and red represents dead bacterial cells within the biofilms. The third column in each group is a merge of the previous two columns (live and dead stain).

SUPPLEMENTARY FIGURE 3 Killing of MRSA biofilm formation on the glass surface of confocal dish culture plate at days 1 **(A)** and 3 **(B)** by fluorescence microscopy. The biofilms were stained with live and dead stains, where green represents live and red represents dead bacterial cells within the biofilms. The third column in each group is a merge of the previous two columns (live and dead stain).
