# Supplementary figures and images for: Antimicrobial potency, prevention ability, and killing efficacy of daptomycin-loaded versus vancomycin-loaded β-tricalcium phosphate/calcium sulfate for methicillin-resistant Staphylococcus aureus biofilms
Source: Front Microbiol. 2022 Nov 3;13:1029261. doi: 10.3389/fmicb.2022.1029261 (PMC9669593; doi:10.3389/fmicb.2022.1029261)

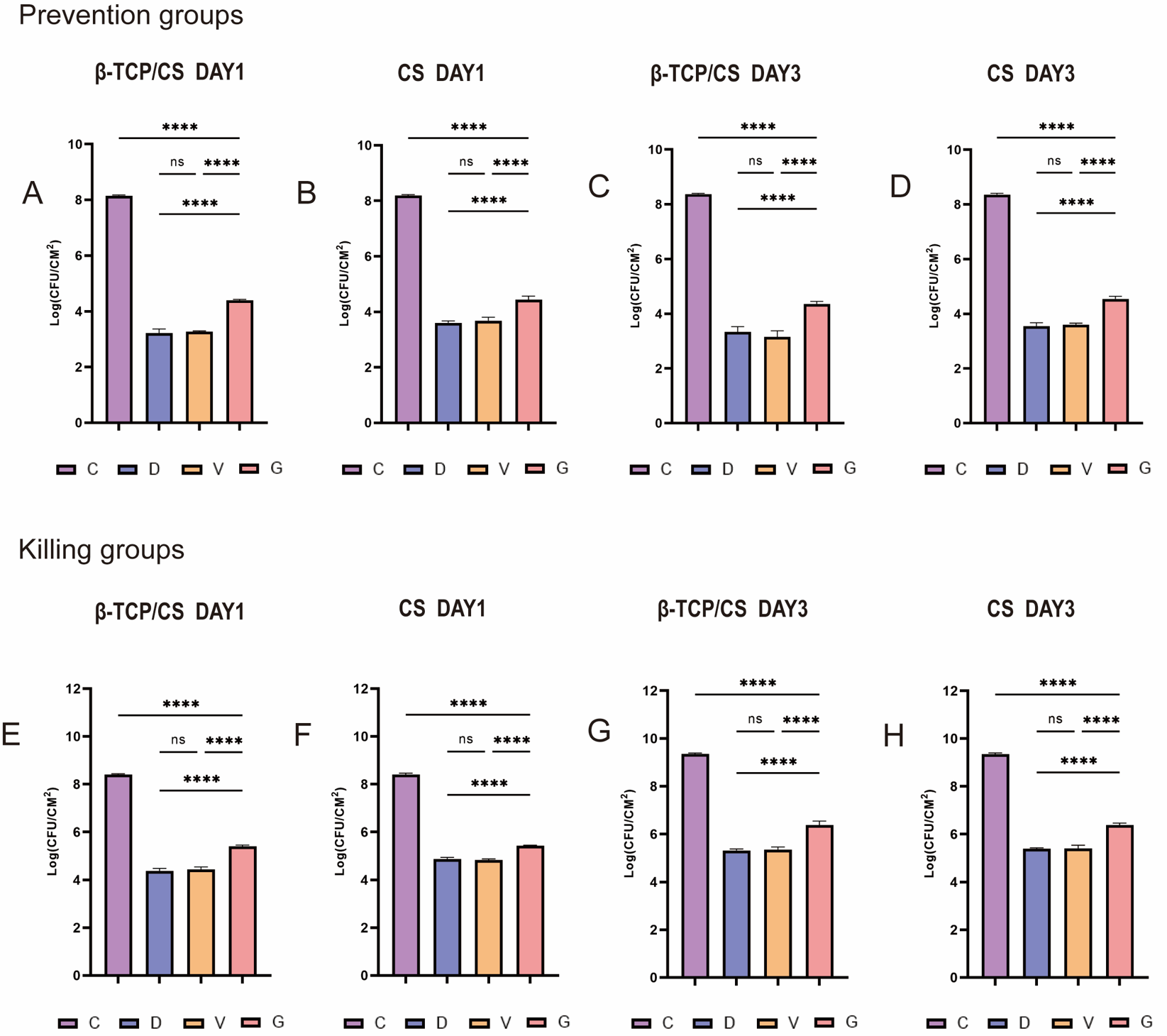

Supplement: SUPPLEMENTARY FIGURE 1 — Methicillin-resistant Staphylococcus aureus (MRSA) colony forming units (CFUs) were counted on days 1 and 3 between the prevention and killing groups. The data are expressed as mean ± standard deviation (SD). Prevention groups: (A–D); killing groups: (E–H). C refers to the control group with blank beads (without antibiotics); ns: not significant, ****p < 0.001. [file Image_1.TIF]

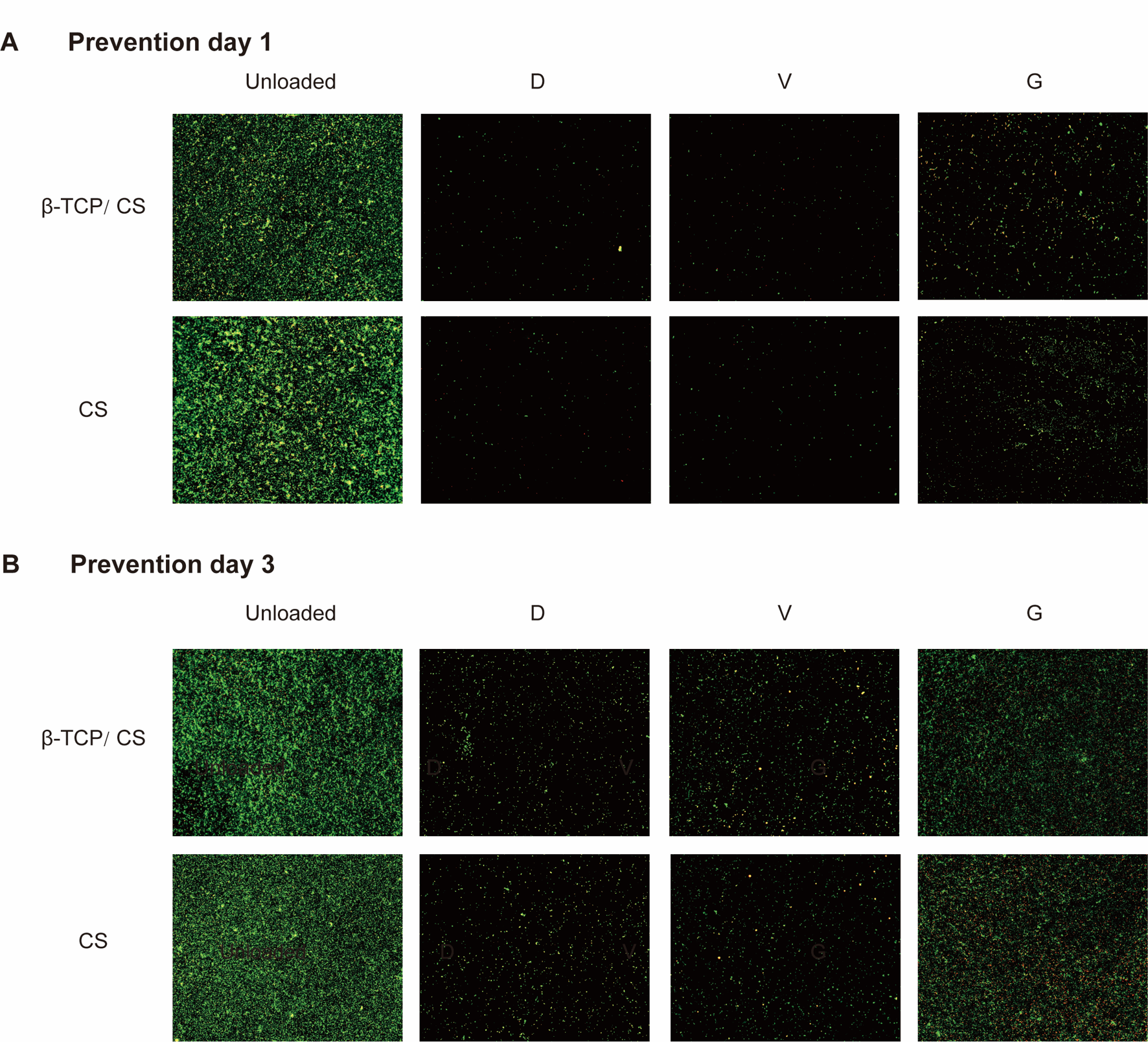

Supplement: SUPPLEMENTARY FIGURE 2 — Prevention of MRSA biofilm formation on the glass surface of confocal dish culture plate at days 1 (A) and 3 (B) by fluorescence microscopy. The biofilms were stained with live and dead stains, where green represents live and red represents dead bacterial cells within the biofilms. The third column in each group is a merge of the previous two columns (live and dead stain). [file Image_2.TIF]

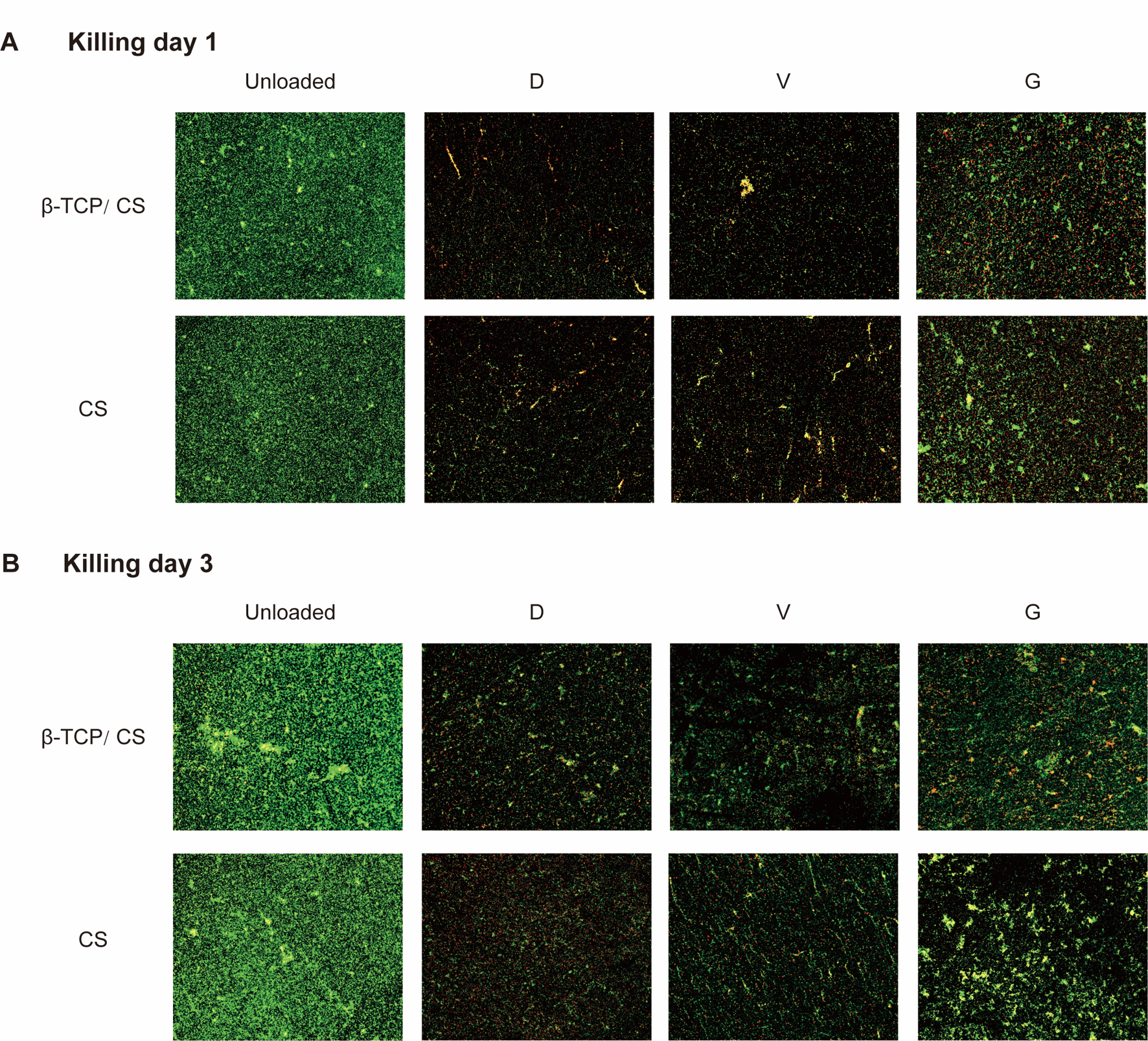

Supplement: SUPPLEMENTARY FIGURE 3 — Killing of MRSA biofilm formation on the glass surface of confocal dish culture plate at days 1 (A) and 3 (B) by fluorescence microscopy. The biofilms were stained with live and dead stains, where green represents live and red represents dead bacterial cells within the biofilms. The third column in each group is a merge of the previous two columns (live and dead stain). [file Image_3.TIF]
